# Cardioprotection by systemic dosing of thymosin beta four following ischemic myocardial injury

**DOI:** 10.3389/fphar.2013.00149

**Published:** 2013-11-29

**Authors:** Weike Bao, Victoria L. Ballard, Saul Needle, Bao Hoang, Stephen C. Lenhard, James R. Tunstead, Beat M. Jucker, Robert N. Willette, G. Teg Pipes

**Affiliations:** ^1^Metabolic Pathways and Cardiovascular Unit, GlaxoSmithKline PharmaceuticalsKing of Prussia, PA, USA; ^2^Platform Technology and Science, GlaxoSmithKline PharmaceuticalsKing of Prussia, PA, USA

**Keywords:** thymosin beta four, myocardial ischemia, ischemia/reperfusion, angiogenesis

## Abstract

Thymosin beta 4 (Tβ4) was previously shown to reduce infarct size and improve contractile performance in chronic myocardial ischemic injury via two phases of action: an acute phase, just after injury, when Tβ4 preserves ischemic myocardium via antiapoptotic or anti-inflammatory mechanisms; and a chronic phase, when Tβ4 activates the growth of vascular or cardiac progenitor cells. In order to differentiate between the effects of Tβ4 during the acute and during the chronic phases, and also in order to obtain detailed hemodynamic and biomarker data on the effects of Tβ4 treatment suitable for use in clinical studies, we tested Tβ4 in a rat model of chronic myocardial ischemia using two dosing regimens: short term dosing (Tβ4 administered only during the first 3 days following injury), and long term dosing (Tβ4 administered during the first 3 days following injury and also every third day until the end of the study). Tβ4 administered throughout the study reduced infarct size and resulted in significant improvements in hemodynamic performance; however, chamber volumes and ejection fractions were not significantly improved. Tβ4 administered only during the first 3 days following injury tended to reduce infarct size, chamber volumes and improve hemodynamic performance. Plasma biomarkers of myocyte injury were significantly reduced by Tβ4 treatment during the acute injury period, and plasma ANP levels were significantly reduced in both dosing groups. Surprisingly, neither acute nor chronic Tβ4 treatment significantly increased blood vessel density in peri-infarct regions. These results suggest the following: repeated dosing may be required to achieve clinically measureable improvements in cardiac function post-myocardial infarction (MI); improvement in cardiac function may be observed in the absence of a high degree of angiogenesis; and that plasma biomarkers of cardiac function and myocardial injury are sensitive pharmacodynamic biomarkers of the effects of Tβ4.

## Introduction

Thymosin beta 4 (Tβ4) is a widely-expressed peptide which has been shown to regulate multiple cellular processes, including cell migration (Malinda et al., 1997; Kobayashi et al., 2002; Sosne et al., 2002; Bock-Marquette et al., 2004), survival (Bock-Marquette et al., 2004; Sosne et al., 2004) and differentiation (Huang et al., 2006; Philp et al., 2007). Tβ4 has also been implicated in wound healing in a range of organs (Goldstein et al., 2012; Sosne et al., 2012) as well as regenerative processes, most notably vasculogenesis associated with endothelial progenitor cells (Hinkel et al., 2010). In the heart, Tβ4 has been suggested to play a role in cardioprotective mechanisms following ischemic injury. Bock-Marquette et al. reported that Tβ4 treatment initiated just after permanent ligation of the left anterior descending (LAD) coronary artery in mice resulted in improved ejection fraction 14 and 28 days post-surgery; additionally, scar volume was reduced 28 days post-surgery (Bock-Marquette et al., 2004). This was reported to be due to preservation of ischemic cardiac myocytes, potentially via activation of the pro-survival Akt pathway. A subsequent study using the same mouse model found significant increases in coronary blood vessel growth following Tβ4 treatment (Bock-Marquette et al., 2009). Additional mechanistic studies in a 7-days mouse myocardial infarction (MI) model indicated that the PIP complex activates Akt in this setting (Sopko et al., 2011). Tβ4 was reported to have qualitatively similar effects in large animals: retroperfusion of Tβ4 increased cardiomyocyte survival in a pig ischemia-reperfusion model of cardiac injury, with concomitant improvements in two measures of cardiac contractility, subendocardial segment shortening and dP/dtmax (Hinkel et al., 2008).

Tβ4 was previously shown to dramatically reduce myocardial injury following only one dose, administered directly to the heart just after injury, by direct intra-cardiac injection (Bock-Marquette et al., 2004; Sopko et al., 2011) and by coronary retroperfusion (Hinkel et al., 2008). Because systemic administration of Tβ4 would be safer and more convenient than direct cardiac injection or retroperfusion, we measured the cardioprotection resulting from repeated dosing with Tβ4 systemically, every day for the 3 days period immediately following the onset of ischemia. For comparison, we also tested repeated dosing every day for the first 3 days followed by dosing every third day until the end of the study. Lastly, in order to verify that Tβ4 treatment would have clinical utility in the setting of ischemia-reperfusion in addition to permanent myocardial ischemia, we tested the effects of repeated IP Tβ4 in an ischemia-reperfusion model.

These studies were performed in the rat, which is larger and has more consistent coronary vessel anatomy than the mouse. The increased size of the rat provides more material for histological, biochemical and plasma biomarker quantification, and facilitates detailed measurements of cardiac physiology, potentially providing *in vivo* pharmacodynamic readouts of Tβ4 efficacy. Additionally, the more consistent coronary vessel anatomy in the rat facilitates induction of similarly-sized infarcts using coronary artery ligation.

## Methods

All studies were conducted in accordance with the GSK Policy on the Care, Welfare and Treatment of Laboratory Animals and were reviewed the Institutional Animal Care and Use Committee either at GSK or by the ethical review process at the institution where the work was performed.

### Permanent occlusion model

After anesthesia with Nembutal [60 mg/kg, intraperitoneal (IP) injection], male Sprague-Dawley rats (250–300 g, Charles River, Raleigh, NC) were intubated with polyethylene-190 tube and ventilated with a small animal volume-controlled respirator with a tidal volume of 10 ml/kg at 90 cycles/min (Harvard Apparatus, Holliston, MA). Rats were placed in a supine position on a heated rat surgical table (Harvard Apparatus, Holliston, MA) to prevent hypothermia during anesthesia and surgery. The heart was exposed via sternotomy with use of a small retractor. A 7-0 suture was passed under the left anterior descending coronary artery (LAD) 1 mm below the left atrium for permanent ligation of the LAD artery. The incision was closed by layers using 5-0 suture. The endotracheal tube was removed after spontaneous breathing recovered. Sham-operated animals underwent a similar surgical procedure with the exception of LAD artery ligation. Tβ4 was supplied by RegeneRx Biopharmaceuticals, Inc. (lot # FTHYB40602B). Tβ4 was dissolved in sterile water (HOSPIRA, Inc., Lake Forest, IL) for injection to make a solution with a Tβ4 concentration of 1.074 mg/ml. Tβ4 or vehicle (sterile water for injection) was administered immediately after MI. Rats were injected with either 5 mL/kg of vehicle or 5.37 mg/kg Tβ4 in vehicle, intra-peritoneally (IP). One treatment group received Tβ4 immediately following surgery and for the 2 days following, then additional Tβ4 every third day (long-term dosing). A second treatment group received Tβ4 immediately following surgery and for the 2 days following only (short term dosing). Plasma samples were drawn 3 days after the surgery, then at 28 days (at the end of the study). Cardiac function and structure was evaluated at 14 and 28 days by MRI.

### Ischemia-reperfusion model

After anesthesia with Nembutal (60 mg/kg, IP injection), male Sprague-Dawley rats (250–300 g) were intubated with a polyethylene-190 tube and ventilated with a small animal volume-controlled respirator with a tidal volume of 10 ml/kg at 90 cycles/min (Harvard Apparatus, Holliston, MA). Rats were placed in a supine position on a heated rat surgical table (Harvard Apparatus, Holliston, MA) to prevent hypothermia during anesthesia and surgery. The heart was exposed via sternotomy with use of a small retractor. A 7-0 suture was passed under the LAD 1 mm below the left atrium and tied to a non-traumatic balloon occluder for occlusion and reperfusion of that artery. LAD occlusion and reperfusion was induced by inflating and deflating the balloon occluder. Successful performance of coronary occlusion and reperfusion was verified by color change in the apex, by observing S-T segment elevation and widening of the QRS by ECG (ADInstruments, Colorado Springs, CO) during ischemia, and their resolution after reperfusion. The incision was closed by layers using 5-0 suture. The endotracheal tube was removed after spontaneous breathing recovered. Rats were subjected to 30 min of myocardial ischemia followed by 23.5 h of reperfusion injury, then analyzed for infarct size and area at risk. Rats were injected with either 5 mL/kg of vehicle or 5.37 mg/kg of Tβ4 in vehicle, IP, during the ischemic period, then 2 h after initiation of reperfusion. Two separate groups of five rats each were subjected to 30 min of ischemia followed by 4 h of reperfusion, then harvested for Akt analyses; these rats were injected with either 5 mL/kg of vehicle or 5.37 mg/kg of Tβ4 in vehicle, IP, during the ischemic period, then 2 h after initiation of reperfusion.

### Hemodynamic measurements and post-mortem analyses

At the end of study, a 2F Millar Mikro-tip catheter transducer was inserted into the left ventricle through the right carotid artery to measure left ventricular pressure and +dP/dtmax and—dP/dtmin. Heart and lung weights were measured after invasive hemodynamic measurements. Hearts were perfused and fixed with 10% neutral phosphate-buffered formalin (10%NBF) at a pressure of 90 mmHg, then were kept in 10% NBF for 16–18 h before transferring to 70% ethanol for histology analysis. Hemodynamic measurements on one animal from the short term dosing group could not be captured due to vessel rupture.

### Cardiac MRI

Rats were anesthetized using 1.5–2.0% isoflurane (1 L/min), and placed inside of a radio frequency coil (ID 11.6 cm) for MR imaging in a 9.4T horizontal bore magnet (Bruker, Billerica, MA). A tri-pilot Intragate FLASH scout image was acquired using the following parameters: TE/TR = 1.3/128 ms, FOV = 60 mm × 60 mm, #reps = 8, 128 matrix, slice thickness = 1 mm, 469 micron in plane resolution, 10 slices/orientation, TA = 1 min 5 s. Coronal and sagital long axis Intragate FLASH images were acquired using the following parameters: TE/TR = 2.06/9.1 ms, FOV = 50 mm × 50 mm, #reps = 160, 128 matrix, slice thickness = 2 mm, 391 micron in plane resolution, TA = 93 s. A series of 2 mm thick short axis (axial) images (same parameters as long axis images) were acquired through the entire left ventricle of the rat heart (~7–8 slices). Cardiac image analysis was performed using Analyze 8.1 AVW software (AnalyzeDirect, Lenexa KS). Axial images of the left ventricle were reconstructed to 512 × 512 pixels prior to the manual tracing of the epicardial and endocardial borders of the left ventricle in both diastole and systole. The summation of the individual axial slice volumes for the left ventricle tissue (in diastole), ED lumen volume and ES lumen volume allowed for the quantification of LV mass, EDV, ESV, and Ejection Fraction (EF%). The formulas for the cardiac indices are: LV Mass (mg) = LV Volume * 1.05 (density of cardiac tissue); EDV (ul) = Sum of all axial lumen volumes in diastole; ESV (ul) = Sum of all axial lumen volumes in systole; SV (ul) = (EDV-ESV); EF% = (EDVESV)/EDV * 100.

### Plasma biomarker quantification

250 uL of whole blood was collected in EDTA via tail vein. Blood samples were centrifuged and plasma collected and stored at −80° until tested. Cardiac troponins, myosin light chain and FABP3 were quantified using a cardiac injury protein quantification kit (Meso Scale Discovery, Rockville, MD, # K15161C-1) using manufacturer's protocols. Pro-ANP levels were quantified using an ELISA kit (catalog # 04-BI-20892) from ALPCO Diagnostics (Salem, MA) using manufacturer's protocols.

### Determination of infarct size (permanent occlusion model)

Transverse 2 mm rings of cardiac left ventricles were formalin fixed and processed for paraffin embedding. A 5 μm section from each ring, representing distances of 2, 4, 6, and 8 mm from the apex, were stained with Masson Trichrome for quantitative analysis of infarct size. Each ring was photographed, and infarcted regions of the left ventricle were defined as those bordering blue staining. For calculation of infarct volumes, the number of blue pixels in the infarct regions from all four sections were added together, then normalized to overall ring area from all four sections (red + blue pixels). One apical ring with no discernible lumen was excluded from the short term dosing group. One MI-operated animal with no evidence of infarct from the short term dosing group was also excluded from all analyses.

### Blood vessel density analysis

Automated vessel/unit area ratio counts of rat endothelial cell antigen positive vessels were averaged from four regions of interest exhibiting primarily transverse cross-sections of muscle fibers within the proximity of the infarct. Regions of interest were preferentially selected from sections 6 then 4 mm from the apex and from non-transmural in preference to transmural infarct areas. The anti-RECA antibody used was clone HIS52 (Serotec, Raleigh, NC).

### Determination of infarct size/area at risk (ischemia-reperfusion model)

After hemodynamic measurements, the heart was excised and perfused with saline to wash out residual blood through an aortic cannula (18-gauge needle). To delineate infarcted tissues from viable myocardium, the heart was then perfused with a 1% solution of 2,3,5-triphenyltetrazolium chloride (TTC) in phosphate buffer (pH 7.4, 37°C). The viable myocardium stained red, and the infarcted myocardium stained white. To delineate the area at risk (ischemic area), the coronary artery was then tied at the site of the previous occlusion and the aortic root was perfused with a 1% Evans blue dye (Sigma) in normal saline. As a result of this procedure, the portion of the LV supplied by the previously occluded coronary artery (area at risk) was identified by the absence of blue dye, whereas the rest of the LV was stained dark blue. The heart was frozen, after which all atrial and right ventricular tissues were excised. The LV was cut into transverse slices, which were photographed using a digital camera. The borders of the infarct, ischemic and nonischemic area of heart image were traced and measured using Image-Pro Plus and from these measurements infarct size was calculated as a percentage of the ischemic area. Ischemic area was calculated as a percentage of LV area.

### Analysis of Akt phosphorylation

Tissue from rat LV was extracted and stored at −80^°^ after study. Samples (½ rat LV) were placed into tubes submerged in liquid N2 and drained of excess followed by blunt force pulverization. Tissue was lysed with a mild detergent (750 ul) containing phosphatase inhibitors (Bio-Rad Bio-Plex cell lysis buffer, Hercules, CA) vortexed and placed on ice × 15 min with shaking. Tubes were centrifuged at 2000 g × 10 min and supernatant was collected and protein determined by BCS method. Lysates were normalized to 0.2–0.9 mg/ml and analyzed by MSD assay (Meso Scale Discovery, #N45100B-1) for phospho-Akt induction.

### Data analyses

Data are presented as mean ± s.e.m. unless noted otherwise. All other comparisons were made by t-test or one-way ANOVA using GraphPad Prism software v5.0 (GraphPad Software, San Diego, CA), unless otherwise noted.

## Results

### Chronic Tβ4 treatment reduces infarct size and preserves hemodynamic function in the rat permanent lad occlusion model

The first set of experiments tested the effects of Tβ4 in a 28 days, permanent LAD occlusion model of MI in the rat. We compared four groups: sham-operated, MI + vehicle injection, MI + short term dosing of Tβ4 and MI + long term dosing of Tβ4. The short term dosing group received IP Tβ4 injections just after MI surgery, then daily for the subsequent 2 days, at a dose of 5.37 mg/kg body weight (similar to the dose per kg body weight administered to mice in Bock-Marquette et al., 2004). The long term dosing group received the same treatment for the first 3 days following surgery, but then also received IP Tβ4 every 3 days until the end of the study. All animals for this study were sacrificed 28 days after LAD occlusion.

**Figure 1:**
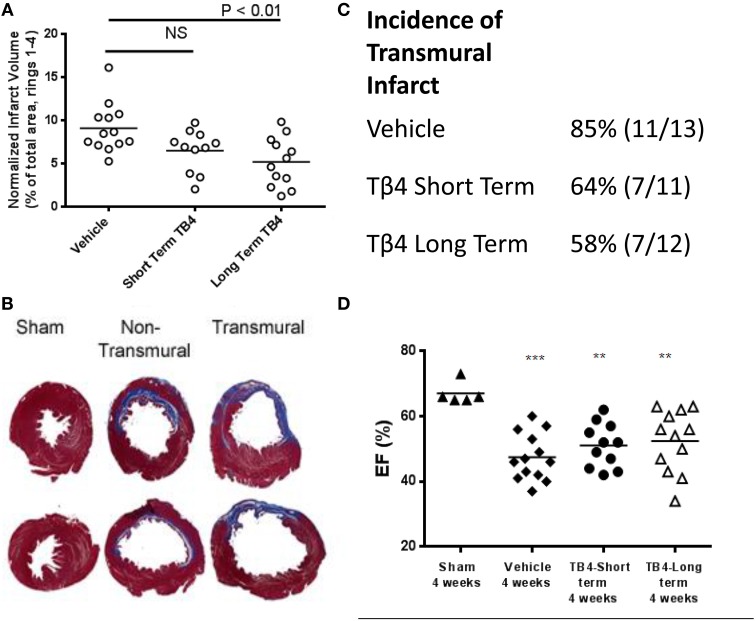
**Tβ4 treatment reduces infarct volume in the rat 28-days permanent LAD occlusion model, but does not significantly improve ejection fraction. (A)** Average infarct volume was significantly reduced by repeated dosing with Tβ4. Short term-dosing with Tβ4 trended toward reduced infarct volume, but this effect did not reach significance. Each point represents one animal, and the horizontal line represents the average for the group. **(B)** Examples of infarcts. Non-transmural infarcts often left a large portion of surviving myocardial tissue surrounding a sub-endocardial infarct. **(C)** Tβ4-treated groups trended toward a reduced incidence of transmural infarct, but this effect did not reach statistical significance (*P* = 0.2, Fischer's test). **(D)** Measurement of %EF at end-of-study revealed trends toward improved function which did not reach significance. %EF was also measured mid-study and effects of MI and TB4 treatment were similar (not shown). Horizontal bars in A and C represent the mean of each group. ^**^P < 0.01 vs. Sham, and ^***^P < 0.001 vs. Sham using a one-way ANOVA and a Tukey's *post-hoc* test.

Histological analysis showed that LAD occlusion resulted in significant loss of myocytes and replacement fibrosis in the infarcted area 28 days following surgery. However, Tβ4 treatment was able to limit this injury: as measured by cross-sectional area of four sections per heart, mean infarct volume was significantly reduced by 43% in the long term dosing group (from 9.1 ± 0.8 to 5.2 ± 0.8%; *P* < 0.01) and by 29% in the short term dosing group, although this did not reach statistical significance (Figure 1). In 11 of the 13 vehicle-treated MI controls (11/13), the infarct was transmural, while in the Tβ4-treatment groups the incidence of transmural infarction tended to be lower (7/11 in the short-term group and 7/12 in the long-term treatment group). Tβ4 treatment had no effect on survival through the peri-operative period: two animals from each group receiving LAD occlusion died on the day following surgery. Survival in all groups from 24 h after surgery to the end of the study was 100%.

LAD occlusion resulted in significant decreases in left ventricular systolic pressure (LVSP), maximum dP/dt, and minimum dP/dt, and increases in left ventricular end diastolic pressure (LVEDP) (Table 1). Long-term dosing with Tβ4 was associated with significant improvements in LVEDP, maximum dP/dt and minimum dP/dt, demonstrating a preservation of hemodynamic function. The short term Tβ4 dosing group displayed trends toward improved hemodynamic measurements, but these effects were not significant.

**Table 1:** **Cardiac function 4 weeks after myocardial infarction**.

	**Sham**	**MI + Vehicle**	**MI + Tβ4-short term dosing**	**MI + Tβ4-long term dosing**
*N*	5	13	10	12
Left ventricular systolic pressure (mm Hg)	122±8	90±3^**^	103±2.6^*^	113±3^#^
Left ventricular end diastolic pressure (mm Hg)	5.3±1.31	10.3±0.98^**^	7.7±0.46	7.1±0.74^#^
Heart rate (bpm)	376±17	349±11	344±9	366±8
dP/dt_max_ (mmHgs^-1^)	8799±505	5701±304^***^	6609±251^**^	7704±412^†^
dP/dt_min_ (mmHgs^-1^)	8157±725	4972±263^**^	5459±268^**^	6638±301 ^§^
EDV (uL)	485±23	784±43^***^	703±49^**^	699±43^**^
ESV (uL)	158±11	419±34	350±34^*^	344±41^*^
EF%	67.2±1.5	47.4±2.0^***^	51±2.01^***^	52.4±2.7^***^

Data are mean ± s.e.m. Tβ4-short term dosing rats were treated with 5 mg/kg/day Tβ4IP for 3 days post-MI, with the first dose administered immediately post-MI. Tβ4-long term dosing rats were treated with 5 mg/kg/day Tβ4IP for the first 3 days, then every third day up to the end of the study on day 28, with the first dose administered immediately post-MI.

* p < 0.05 vs. sham;

** p < 0.01 vs. sham;

*** p < 0.001 vs. sham;

# p < 0.05 vs. vehicle;

§ p < 0.01 vs. vehicle;

† p < 0.00 vs. vehicle.

Ejection fractions were reduced in all MI groups, in comparison to sham-operated animals. The reduction in infarct volumes in the long term Tβ4 dosed group translated into a non-significant 5% increase in ejection fraction at 28 days (Table 1 and Figure 1). The decrement in systolic blood pressure induced by MI was partly attenuated by longterm Tβ4 treatment.

**Figure 2:**
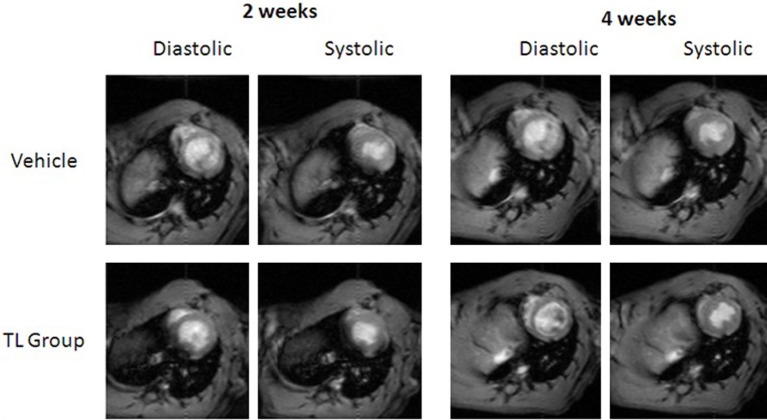
**MRI images showing similar cardiac structure at systole and diastole, from mid-study (2 weeks) and at end of study (4 weeks), among TB4-treated (bottom row) and vehicle-treated animals (top row)**. Images shown are representative images taken from the same animals at the indicated time points.

Additionally, transient benefits of Tβ4 treatment on cardiac function were not observed. Analyses of MRI images taken at 14 days post-occlusion and 28 days postocclusion revealed no other significant differences in EDV, ESV, septal wall thickness, anterior wall thickness or posterior wall thickness among the groups receiving MI surgery (Figure 2 and data not shown).

### Biomarkers of myocardial damage are reduced by Tβ4 treatment during the acute phase of permanent myocardial ischemia

**Figure 3:**
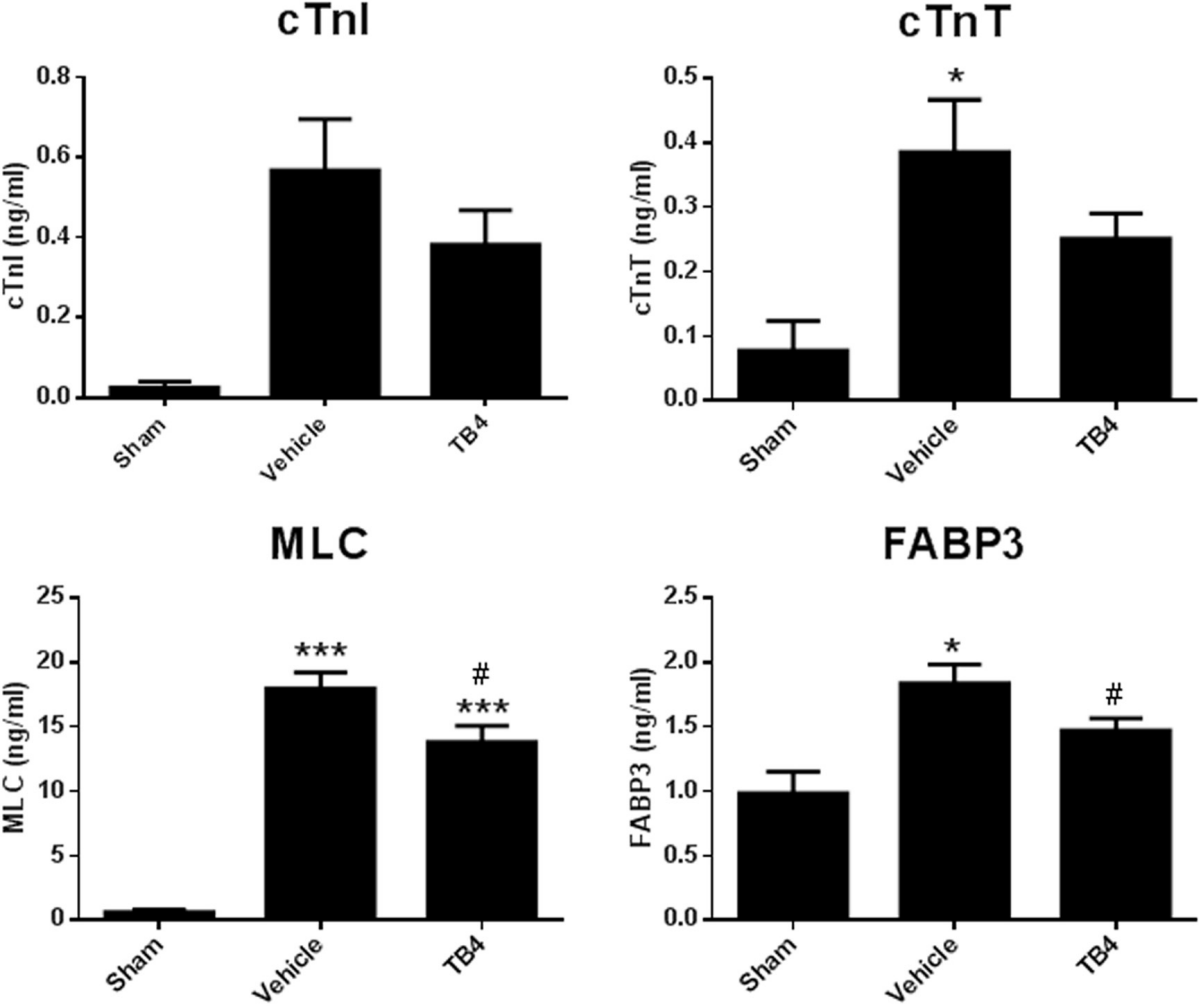
**Plasma levels of markers of myocyte damage 3 days post-MI surgery**. Elevations in cTnI due to MI were highly variable and did not reach statistical significance. Elevation in cTnT due to MI was significant, but the trend toward reduction in the MI group treated with Tβ4 was not. Elevation in MLC and FABP3 due to MI were significantly reduced in the Tβ4 treatment groups. Note that at the 3 days time point, dosing regimens were identical between the long term treatment and short term treatment groups; accordingly, data from both Tβ4 dosing regimens have been combined into one group for analysis. ^*^P < 0.05 vs. Sham using a one-way ANOVA and a Tukey's *post-hoc test*; ^***^ *P* < 0.001 vs. Sham using a one-way ANOVA and a Tukey's *post-hoc* test; ^#^*P* < 0.05 vs. MI + Vehicle using a one-way ANOVA and a Boneferroni's *post-hoc* test.

Plasma biomarkers of myocardial injury were measured at 3 days post-surgical injury to determine whether Tβ4 administration prevented myocyte loss during the acute period, just following MI. At this time point, animals in both the long-term and the short-term Tβ4 dosing groups had received identical doses of Tβ4; therefore the two Tβ4 treatment groups were combined for analysis. Plasma levels of MLC and FABP3 were significantly elevated 3 days post MI, and this effect was attenuated by Tβ4 treatment. Trends toward reduced levels of cardiac troponins were observed, but these effects were not significant. Additionally, cardiac troponin I appeared elevated in the MI group, but this elevation was not statistically significant (Figure 3).

### Tβ4 attenuates post-infarct cardiac hypertrophy

**Figure 4:**
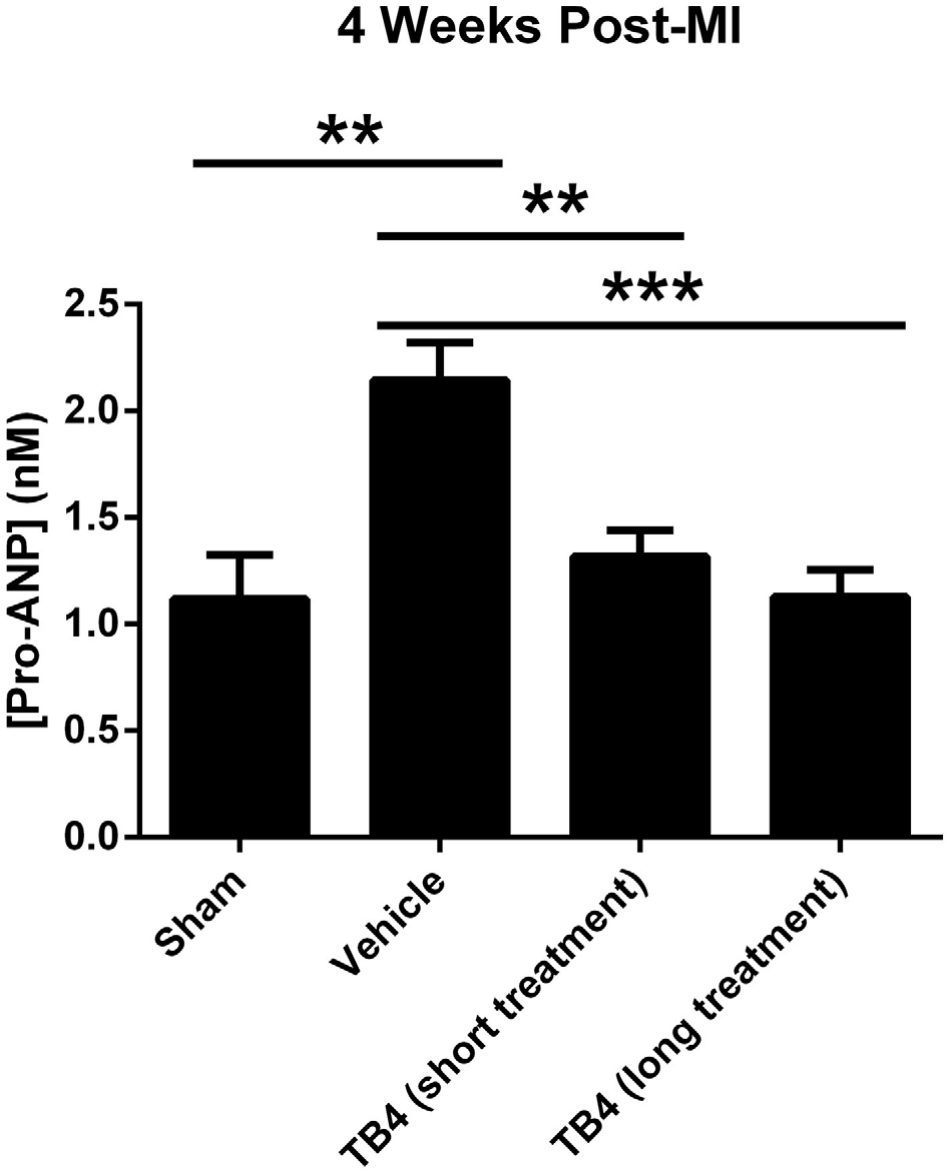
**Plasma pro-ANP levels 28 days post-MI surgery**. Tβ4 treatment significantly reduced plasma pro-ANP levels 28 days after MI surgery. ^**^P < 0.01, Sham vs. MI + Vehicle; ^**^P < 0.01, MI + Vehicle vs. Tβ4 (short treatment); ^***^P < 0.001, MI + Vehicle vs. Tβ 4 (long treatment).

Tβ4 treatment tended to reduce gravimetric measurements of cardiac hypertrophy (Table 2). This effect was small, reaching statistical significance only in the long-term dosing group. Right ventricular weights were significantly reduced in the long-term dosing group. Consistent with these findings, treatment with Tβ4 reduced plasma pro-ANP levels in both short-term and long-term treatment groups (Figure 4). Notably, plasma pro-ANP levels were almost as low in the Tβ 4-treated groups as in the sham operated control group at 28 days.

**Table 2:** **Organ weights in Sprague-Dawley rats 4 weeks after myocardial infarction**.

	**Sham**	**MI + Vehicle**	**MI + Tβ4 short term dosing**	**MI + Tβ4 long term dosing**
*N*	5	13	11	12
Body weight (g)	387±17	387±11	389±12	377±7
Heart weight (g)	1.1±0.05	1.3±0.05^*^	1.2±0.04	1.2±0.03^#^
Right ventricular weight (g)	0.18±0.02	0.24±0.01^*^	0.21±0.01	0.18±0.01^#^
Left ventricular weight (g)	0.88±0.04	1.01±0.03	0.95±0.03	0.95±0.02
Tibial Length (mm)	42.1±0.67	42.0±0.29	42.0±0.19	42.1±0.17
Heart weight/body weight (mg/g)	2.9±0.11	3.5±0.10^*^	3.1±0.08^#^	3.2±0.07
Right ventricular weight/body weight (mg/g)	0.47±0.04	0.61±0.03^*^	0.53±0.01	0.48±0.02^#^
Left ventricular weight/body weight (mg/g)	2.29±0.11	2.62±0.06	2.46±0.07	2.51±0.06
Heart weight/tibial length (mg/mm)	27.0±1.28	31.7±0.98^*^	28.8±0.96	28.5±0.61^#^
Right ventricular weight/tibial length (mg/mm)	4.3±0.39	5.6±0.30^*^	4.9±0.19	4.3±0.21^#^
Left ventricular weight/tibial length (mg/mm)	21.0±1.05	24.0±0.67	22.7±0.80	22.5±0.51

Data are mean ± s.e.m. Tβ4-short term dosing rats were treated with 5 mg/kg/day Tβ4 IP for 3 days post-MI, with the first dose administered immediately post-MI. Tβ4-long term dosing rats were treated with 5 mg/kg/day Tβ4 IP for the first 3 days, then every third day up to the end of the study on day 28, with the first dose administered immediately post-MI. Data are mean ± s.e.m.

# p < 0.05 MI + Vehicle vs. MI + Tβ4 group.

* p < 0.05 Sham vs. MI + Vehicle.

### Blood vessel density is not increased by Tβ4 treatment in the periinfarct region

**Figure 5:**
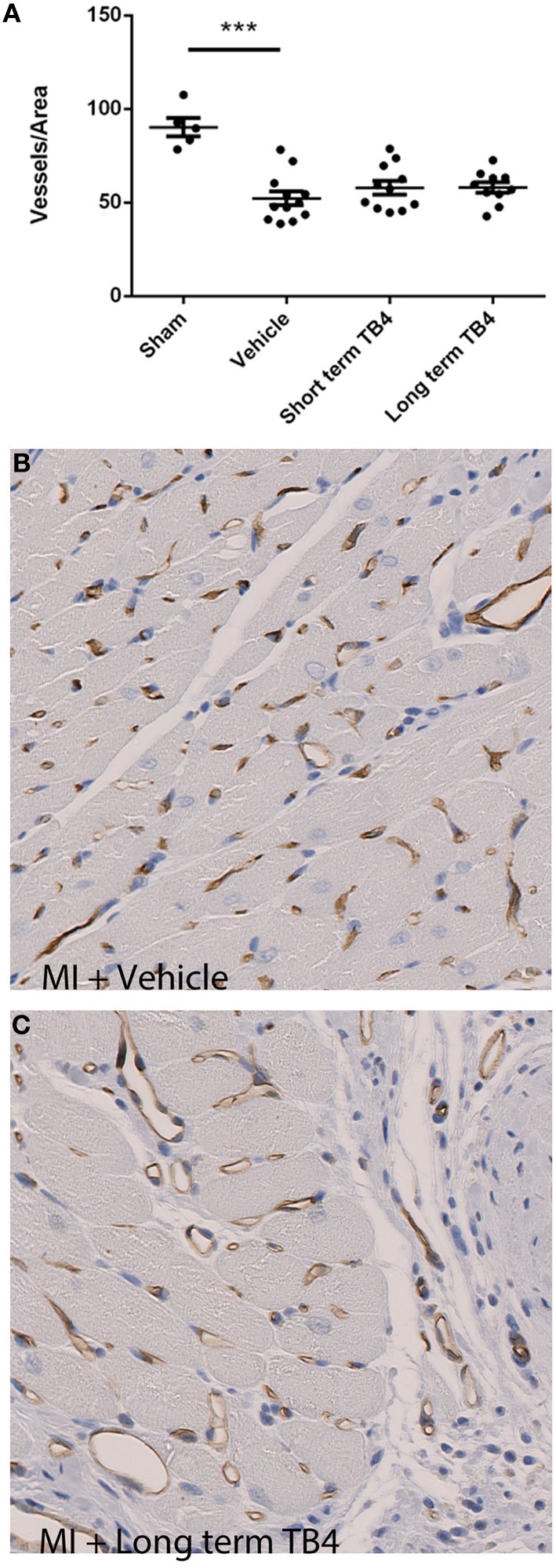
**Quantification of blood vessel density in regions near the infarcts in animals receiving coronary artery ligations. (A)** Tβ4 treated groups did not display a significant increase in blood vessel density per unit area. ^***^P < 0.001 sham operated vs. MI + Vehicle **(B,C)** Representative histological stains for blood vessels from treatment and control sections; brown staining indicates RECApositive staining.

Previous studies indicated that Tβ4 functions partly through a pro-angiogenic mechanism (Smart et al., 2007; Bock-Marquette et al., 2009). In order to determine whether Tβ4 stimulates angiogenesis in the rat MI model, we quantified blood vessel density in regions bordering the infarct. LAD occlusion resulted in significant blood vessel rarefaction. Tβ4 treated groups did not have a significant increase in border zone coronary capillaries in comparison to the vehicle-treated MI control group (Figure 5). Blood vessel densities were not measured mid-study, and so we cannot rule out the possibility of that Tβ4 treatment increases blood vessel density transiently; however, as noted above, mid-study measurements of cardiac structure and function did not reveal significant benefits of Tβ4 treatment.

### Reduction in acute reperfusion injury by Tβ4

**Figure 6:**
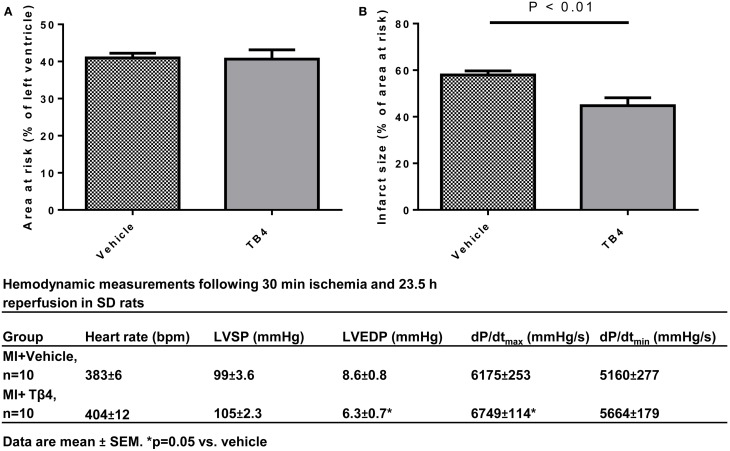
**Tβ4 treatment reduced infarct size in a rat ischemia-reperfusion model of myocardial injury. (A)** Quantification of area-at-risk and infarct sizes in the hearts from rats treated with vehicle vs. rats treated with Tβ 4 administered both during and after the ischemic period. Acute treatment with Tβ 4 did not affect the area at risk. Tβ4 treatment significantly reduced infarct sizes at 23.5 h postischemic injury. **(B)** Table describing hemodynamic measurements at end-of-study. End-diastolic pressures were reduced and dP/dtmax was increased in the Tβ4-treated group in comparison to the vehicle-treated group.

**Figure 7:**
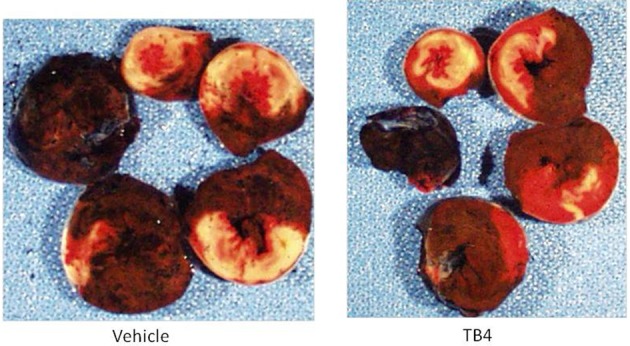
**TB4 treatment reduces infarct sizes without affecting area-at-risk in the acute ischemia-reperfusion model in the rat**. Shown are representative hearts from a treatment and a control animal, sectioned from apex to base (clockwise). White areas represent infarcted tissue; red areas are viable, and blue tissue was not at risk of infarct. The heart from the TB4-treated animal (right panel) shows reduced infarct sizes at multiple levels in comparison to the heart from the vehicle-treated animal.

Tβ4 treatment was previously shown to activate anti-oxidant and anti-apoptotic mechanisms *in vitro* (Wei et al., 2012). In order to evaluate the cardioprotective effects of systemic Tβ4 in an ischemia-reperfusion model (which subjects cardiac myocytes to oxidative stress during the re-perfusion phase,) two groups of 10 rats each were subjected to 30 min of myocardial ischemia, followed by 23.5 h of reperfusion. One group received 5.4 mg/kg Tβ4 IP during the ischemic period and also 2 h later, while the other group received only water. Tβ4 treated animals had significantly reduced infarct size as a percentage of area at risk in comparison to the vehicle-treated control animals (57.9 ± 1.73% in the vehicle-treated animals, compared to 44.7 ± 3.41% in the Tβ4-treated animals; *p* < 0.01; Figure 6). As in the permanent ischemia model, hemodynamic measurements of Tβ4-treated animals demonstrated significantly preserved LVEDP and maximum dP/dt (Figure 6, lower panel). Representative infarct area measurements are shown in Figure 7.

### Activation of Akt signaling in injured myocardial tissue by Tβ4

**Figure 8:**
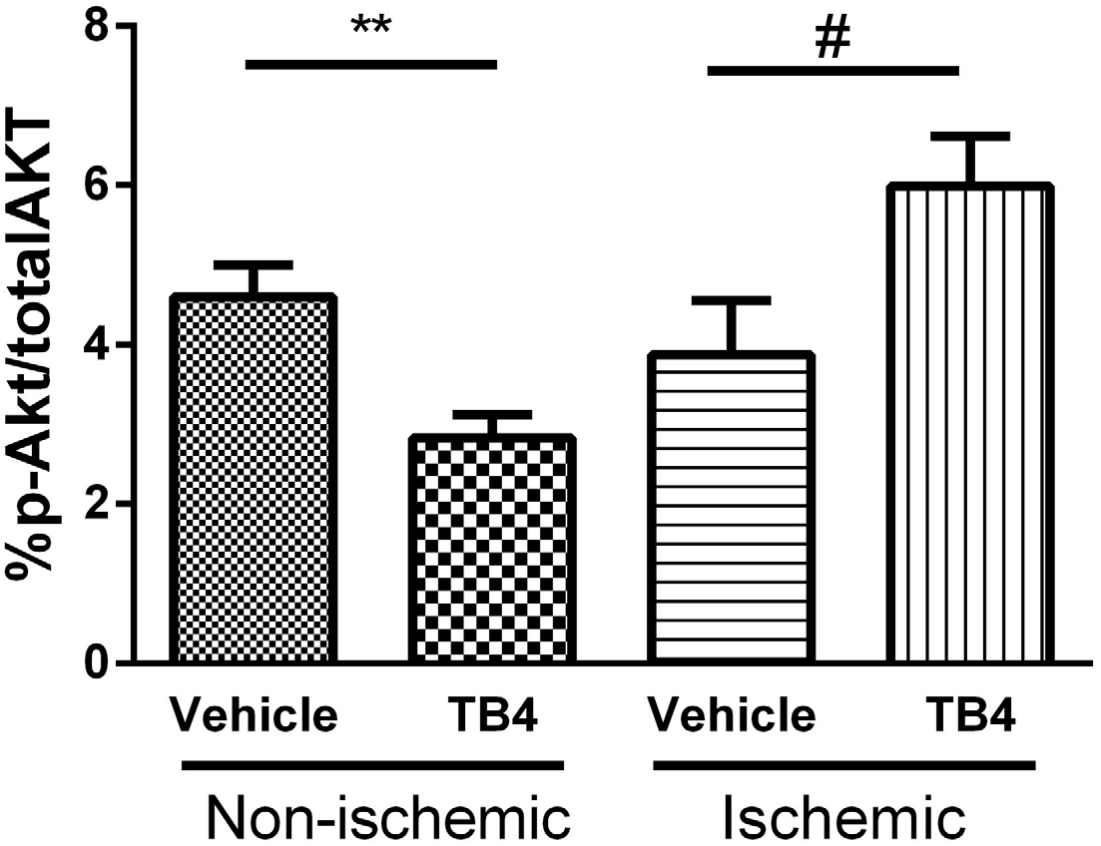
**Activation of the Akt pathway by acute administration of IP Tβ4 in the rat ischemia-reperfusion model was significant but modest, from 4 to 6% in post-ischemic tissues**. Akt activation was reduced in non-ischemic remote myocardial tissue from the same hearts. ^**^P < 0.01 Vehicle (non-ischemic) vs. Tβ4 treated (non-ischemic); ^#^*P* < 0.05 Vehicle (ischemic) vs. Tβ4 treated (ischemic).

Activation of Akt signaling during the acute phase of ischemic injury has been proposed as the mechanism by which Tβ4 administration protects myocardial tissue during ischemia (Bock-Marquette et al., 2004). We assessed the level of activation of the Akt signaling pathway during the acute phase of reperfusion injury. At 2.5 h post-surgery, 30 min following the second dose of Tβ4, ischemic and remote LV tissue pieces were isolated. The percentage of Akt present in the active, phosphorylated form was modest in all groups, ranging from 3–5% of total Akt. The percentage of phospho-Akt in Tβ4 treated hearts was significantly elevated in ischemic tissue, but was suppressed in tissue isolated from non-ischemic regions of the same hearts, compared to vehicle-treated controls (Figure 8).

## Discussion

Previous studies performed in the mouse found the following: 1-the majority of cardiomyocyte apoptosis induced by permanent ischemia occurs within 3 days (based on the observation that the number of apoptotic nuclei was similar between sham-operated and coronary artery occlusion-operated mice 3 days after surgery); and, 2-Tβ4 treatment significantly reduced the number of apoptotic cardiomyocytes 24 h post-occlusion, indicating that one mechanism by which Tβ4 reduced infarct size was by reducing cardiomyocyte apoptosis. In the rat, the majority of cardiomyocyte cell loss following coronary artery permanent ligation occurs within the first day after occlusion (Anversa et al., 1998). Therefore, we hypothesized that repeated systemic treatment with Tβ4 during the 3 days period immediately following occlusion would be sufficient to provide maximal preservation of cardiomyocyte number and cardiac function. Three days after coronary occlusion, plasma levels of MLC and FABP3 were significantly increased, and this increase was significantly attenuated by Tβ4 treatment. Therefore, part of the beneficial effects of Tβ4 can be attributed to cardioprotection during the acute phase of cardiomyocyte cell death immediately following ischemic injury. (The levels of cTnI were highly variable at this time point: cTnI levels were not significantly induced even comparing the sham-operated group to the coronary artery occlusion + vehicle treated group).

However, continued dosing of Tβ4 was required in order to obtain significant preservation of cardiac function and reduction in infarct volumes measured 28 days post-occlusion. Specifically, systolic BP, diastolic BP and maximum and minimum dP/dt were all significantly preserved by long-term Tβ4 treatment. Consistently positive trends in cardiac function were observed in the short term Tβ4 dosing group, but these did not reach statistical significance. Long-term dosing with Tβ4 significantly reduced infarct size in the rat permanent MI model by 43%, roughly consistent with the significant reduction in scar volume previously observed in the mouse. These results are consistent with Tβ4 playing a role in maintaining the structure and function of ischemic myocardium beyond the acute phase of cardiomyocyte cell death. Furthermore, the beneficial effects of continued dosing of Tβ4 did not appear to be due to activation of angiogenesis alone, as the increases in blood vessel density in the infarct border zones of animals treated with Tβ4 were not significant by ANOVA.

Previous studies have demonstrated a pro-angiogenic effect of TB4 treatment in mouse hearts following MI (Bock-Marquette et al., 2009; Sopko et al., 2011). It is not clear why in the present study we did not observe significant cardiac angiogenesis 28 days post-MI following systemic TB4 treatment in the rat. One possible explanation is differences in time post-infarct when capillary densities were measured: previous studies performed in the mouse were terminated at 7 days (Sopko et al., 2011) and 7–14 days (Bock-Marquette et al., 2009), while the present study was terminated at 28 days. It is possible that TB4 treatment caused a transient increase in or preservation of coronary blood vessels. However, if this was the case, this transient increase in blood vessel density was not sufficient to significantly improve %EF measured at day 14.

Some markers of post-infarct hypertrophic remodeling were reduced in the Tβ4 treatment groups: ANF levels 28 days post-infarct were significantly reduced in both Tβ4 dosing groups, as were some measures of heart weight. Although consistently observed using both gravitometric and plasma biomarker analyses, these effects were not statistically significant in all dosing groups, or with all measurements. Furthermore, given the reduction in infarct volumes in these groups, it is not clear from current data whether Tβ4 plays a direct role in reducing hypertrophic responses, or the observed reductions in post-infarct hypertrophy were secondary to reduced infarct sizes.

Tβ4 has been proposed as a treatment for cardiac reperfusion injury (Goldstein et al., 2012). Permanent surgical occlusion of the LAD induces myocardial ischemia only, without re-perfusion injury. As such, permanent LAD occlusion does not serve as a model for myocardial injuries associated with acute coronary syndrome patients following re-canalization. In order to evaluate the potential clinical benefits of Tβ4 treatment in such a setting, we tested the effects of repeated Tβ4 administration during both an ischemic period, and then additional dosing during reperfusion. Tβ4 treatment resulted in a significant decrease in infarct size normalized to area-at-risk, with a concomitant improvement in LVEDP and dP/dtmax. These results are consistent with a previous study in which intracoronary Tβ4 was administered during ischemic cardiac injury in a pig model (Hinkel et al., 2008), but indicate that direct coronary administration of Tβ4 is not required to obtain these therapeutic benefits.

Tβ4 treatment during myocardial injury preserved a degree of cardiac structure and function in multiple surgical models in the rat. Repeated dosing with Tβ4 provided greater benefit than dosing only just after injury. Importantly, data presented in the current study indicate that there may be clinical benefit stemming from systemic administration of Tβ4 during the acute period of cardiac injury, i.e., that retroperfusion or direct intra-cardiac injection may not be required in order to obtain benefit measureable via hemodynamic or plasma biomarker testing.

### Conflict of interest statement

All authors were employees of GlaxoSmithKline at the time this work was performed and own company stock.
